# Frequent isolation of methicillin resistant *Staphylococcus aureus* (MRSA) ST398 among healthy pigs in Portugal

**DOI:** 10.1371/journal.pone.0175340

**Published:** 2017-04-11

**Authors:** Teresa Conceição, Hermínia de Lencastre, Marta Aires-de-Sousa

**Affiliations:** 1 Laboratory of Molecular Genetics, Instituto de Tecnologia Química e Biológica António Xavier (ITQB), Universidade Nova de Lisboa (UNL), Oeiras, Portugal; 2 Laboratory of Microbiology and Infectious Diseases, The Rockefeller University, New York, NY, United States of America; 3 Escola Superior de Saúde da Cruz Vermelha Portuguesa (ESSCVP), Lisboa, Portugal; Amphia Ziekenhuis, NETHERLANDS

## Abstract

**Background:**

Although livestock-associated ST398 methicillin-resistant *Staphylococcus aureus* (MRSA) has been widely reported in different geographic regions, MRSA carriage studies among healthy pigs in Portugal are very limited.

**Methods and findings:**

In total, 101 swine nasal samples from two Portuguese farms were screened for MRSA. In addition five swine workers (including one veterinary and one engineer) and four household members were nasally screened. The isolates were characterized by *spa* typing, SCC*mec* typing and MLST. All isolates were tested for antimicrobial susceptibility, presence of *mecA* and *mecC* genes, and virulence determinants.

MRSA prevalence in swine was 99% (100/101), 80% (4/5) in swine workers and 25% (1/4) in household members. All isolates belonged to ST398 distributed over two *spa* types–t011 (57%) and t108 (42%). SCC*mec* type V was present in most of the isolates (n = 95; 82%) while 21 isolates amplified the *mecA* gene only and were classified as nontypeable. The majority of the isolates were resistant to tetracycline (100%), clindamycin (97%), erythromycin (96%), chloramphenicol (84%) and gentamycin (69%). Notably, 12% showed resistance to quinupristin-dalfopristin (MICs 3–8 μg/mL). Beta-hemolysin (81%) and gamma-hemolysin (74%) were the unique virulence determinants detected. None of the isolates harboured PVL or *mecC* gene.

**Conclusions:**

This study showed a massive occurrence of ST398-MRSA in two independent swine farms, highlighting its establishment among healthy pigs in Portugal.

## Introduction

Although methicillin-resistant *Staphylococcus aureus* (MRSA) is still a major cause of healthcare-associated infections (HA-MRSA and CA-MRSA), livestock-associated MRSA (LA-MRSA) has been increasingly being reported from many parts of the world, including Europe. The main LA-MRSA lineage, ST398, was first reported among pigs in France [1] and was subsequently reported in different European countries, with higher rates in countries with higher densities of pig farming [2]. It is now consensual that human subjects professionally exposed to pigs, namely owners, farmers, veterinarians, and abattoir workers have an increased risk of nasal colonization with ST398-MRSA [3]. In addition, family members that are not in direct contact with pigs are also colonized by MRSA-CC398 although at a lower extent [4].

The nosocomial prevalence of MRSA in Portugal is close to 50% and remains one of the highest in Europe [5]. Public buses were found to be highly contaminated with MRSA and constitute a major MRSA reservoir in the country [6]. MRSA reservoirs in the animal setting in Portugal have been very poorly investigated, namely among healthy pigs [7–9].

The aim of the present study was to assess if healthy pigs constitute a MRSA reservoir in Portugal, by evaluating the prevalence, antimicrobial resistance patterns, and clonal profile of MRSA isolates from two independent farms. In addition, transmission to people professionally exposed to pigs and to their family members was also assessed.

## Material and methods

### Farm setting and study design

Two independent Portuguese swine farms, located in Alentejo and separated by approximately 30 km, were included in the study. These farms are production holdings housing piglets born in Portugal and further delivered to slaughterhouses. About 32 to 33 animals live in 8-12m2 stockyards, according to gender and age. A total of 101 piglets (50 from Farm A and 51 from Farm B) aged 10–11 weeks were randomly selected from different stockyards in each farm, including the same proportion of males and females. In addition, three farmers, the farm veterinary, the animal production engineer as well as four household members and their dog were also nasally screened for the presence of MRSA (Table 1).

**Table 1:** Clonal lineages, antimicrobial resistance and virulence of MRSA isolates recovered from piglets, farm workers and households in the two independent farms, Farm A and Farm B.

Farms	Individuals	Swabs	MRSA		ST398 MRSA lineage		Prevalent antibiogram^b^ (no isolates)	Virulence determinants^c^ (no isolates)
			no individuals (%)	no Isolates^a^	*spa* type (no isolates)	SCC*mec* (no isolates)		
Farm A	Piglets	50	49 (98)	53	t011 (43)	V (43)	Ery, Cly, SXT, Gen, Chr, Q-D* (20);	*hlb*; *hlg*
							Ery, Cly, Gen, Chr, Q-D* (15)	
					t108 (10)	V (8)	Ery, Cly, Cip, Q-D* (3)	*hlb*; *hlg* (7)
						NT (2)	Ery, Cly, Cip, Gen, Q-D* (2)	-
	Farmers	1	1 (100)	1	t011	V	Ery, Cly, Gen, Chr, Q-D*	*hlb*; *hlg*
Farm B	Piglets	51	51 (100)	58	t011 (22)	V (22)	Ery, Cly, Cip, SXT, Chr, Q-D (12);	*hlb*; *hlg*
							Ery, Cly, Cip, SXT, Q-D* (5)	
					t108 (36)	V (20)	Ery, Cly, Gen, Chr, Q-D* (11);	*hlb* (17); *hlg* (12)
							Ery, Cly, SXT, Gen, Chr, Q-D* (6)	
						NT (16)	Ery, Cly, Gen, Chr, Q-D* (9)	*hlb* (2)
	Farmers	2	1 (50)	1	t108	NT	Ery, Cly, Gen, Chr, Q-D*	*-*
Farm A and B	Veterinary	1	1 (100)	1	t108	NT	Cly, Cip, Chr, Q-D*	*-*
	Engineer	1	1 (100)	1	t108	V	Ery, Cly, Q-D*	*hlb*; *hlg*
No direct contact–living outside the farms	Households	4	1 (25)	1	t1184	NT	Cly	*-*
	Pet	1	0 (0)	0	-	-	-	*-*

Q-D*- quinupristin-dalfopristin intermediate resistance by disk diffusion

^a^ ten piglets were colonized with more than one distinct strain each

^b^prevalent antibiogram includes >50% of the isolates. Cip, ciprofloxacin; Chr, chloramphenicol; Cly, clindamycin; Ery, erythromycin; Gen, gentamicin; Q-D, quinupristin-dalfopristin; SXT, trimethoprim-sulfamethoxazole.

^c^ *hlb*—beta-hemolysin gene; *hlg*–gamma-hemolysin gene. *hlb* and/or *hlg* were detected in all isolates except when indicated by the number of positive isolates in brackets.

**Fig 1:**
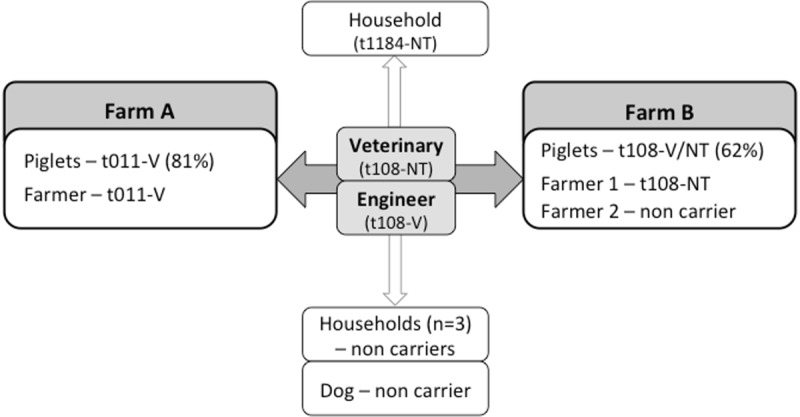
Schematic representation of the interpersonal direct contact between individuals and between individuals and livestock, associated to the distribution of the different MRSA lineages.

None of the household members lived in the swine farms and therefore had no direct contact with the pigs. Among the four household members, three lived with the animal production engineer and the remaining one lived with the veterinary (Fig 1).

### Ethics statement

The protocol was approved by the Research Board of Escola Superior de Saúde da Cruz Vermelha Portuguesa and an oral informed consent was obtained at the time of screening for each human participant.

### Sampling and MRSA identification

Sampling (nasal swabs) was carried out in each farm in a single day in July (Farm A) and in August (Farm B), 2016, respectively. After overnight enrichment growth at 37°C in Mueller-Hinton broth (Becton, Dickinson & Co, New Jersey, USA), the samples were inoculated on Tryptic Soy Agar (TSA) (Becton, Dickinson & Co, New Jersey, USA) and on Chromagar Staph aureus and Chromagar MRSA (ChromAgar, Paris, France). MRSA was confirmed by PCR amplification of the *spa* gene for species identification, and the detection of the *mecA* gene [10,11]. All isolates were additionally tested for the presence of *mecC* [12].

### Antimicrobial susceptibility testing

Antimicrobial susceptibility testing was performed by the disk diffusion method, according to the European Committee on Antimicrobial Susceptibility Testing (EUCAST_ http://www.eucast.org/), for cefoxitin, ciprofloxacin, chloramphenicol, clindamycin, erythromycin, fusidic acid, gentamicin, linezolid, mupirocin, oxacillin, penicillin, quinupristin-dalfopristin (Q-D), rifampin, teicoplanin, tetracycline, trimethoprim-sulfamethoxazole and vancomycin. Q-D minimum inhibitory concentrations (MIC) were determined using E-test strips (Biomerieux, Marcy l'Etoile, France).

### Molecular typing

The isolates were characterized by *spa* typing, multilocus sequence typing (MLST) and SCC*mec* typing as previously described [6]. SCC*mec* V was confirmed by PCR detection of *ccrC* gene [13].

The presence of 11 specific staphylococcal virulence genes, including three leukocidins (*lukS-lukF*, *lukE-lukD*, *lukM*), three hemolysins (*hlb*, *hlg*, *hlgv*) and five super-antigenic toxins (*eta*, *etb*, *etd*, *sel*, *sep*) were determined by multiplex PCR as previously described [14].

### Statistical analysis

Categorical variables were compared using the χ2 or Fisher’s exact test when appropriate, using GraphPad software version 6.0 (GraphPad Software, La Jolla California USA). In all cases, P values of ≤0.05 were considered statistically significant.

## Results and discussion

### MRSA prevalence

Overall, 99% of the piglets (Farm A n = 49 and Farm B n = 51) were nasally colonized with MRSA, and half (5/9; 55.5%) of the humans sampled were MRSA carriers (veterinary, engineer, two out of three farmers and one out of four household members). No differences in MRSA prevalence were observed regarding each farm individually, or by animal gender (p = 0.495 and p = 1.000, respectively). Since 10 piglets were colonized with more than one distinct strain each, a total of 116 MRSA isolates were recovered and subsequently characterized. None of the isolates carried *mecC*.

The frequent isolation of MRSA in pigs (99%) detected in our study is similar to the rates reported by other European countries with high pig farming densities, namely the Netherlands [15]. To the best of our knowledge, the study described in this communication represents the first surveillance of MRSA carriage in pigs and farmer workers in independent large (3000 pigs) pigs production holdings in Portugal. Three independent reports focused on infection and carriage isolates sporadically recovered in Portuguese routine diagnostic laboratories, in two closed-cycle farms of breeding pigs or three farms with around 200 sows, all evidencing a low occurrence of MRSA [7–9].

### Antimicrobial susceptibility

All isolates showed resistance to oxacillin, cefoxitin, penicillin and tetracycline, while the majority was resistant to clindamycin (97%), erythromycin (96%), chloramphenicol (84%) and gentamycin (68%). Resistance to trimethoprim-sulfamethoxazole (44%) and to ciprofloxacin (32%) was also observed. None of the isolates was resistant to rifampicin, fusidic acid, teicoplanin, mupirocin, linezolid or vancomycin. Antimicrobial resistance patterns were similar in both Farm A and Farm B, with the exception of gentamycin resistance, which was more prevalent in Farm A (87.5% *versus* 50% in Farm B; p>0.001).

Tetracyclines, penicillins and sulphonamides are commonly used as feed additives and growth promoters in livestock, including swine in European countries [16], while macrolides and lincosamides are widely used for the treatment of common infections in food-producing animals as cattle and pigs [17], which could support the high resistance levels to these drugs in our isolates. Moreover, in the studied farms, colistin, amoxicillin and zinc oxide are commonly used as feed additives to prevent infections, namely diarrhea.

Notably, 14 isolates (12%) recovered from pigs showed resistance to Q-D with MICs ranging from 3 to 8 μg/mL, while 78% of the entire collection showed an intermediate phenotype. Although resistance to Q-D is rare in staphylococci among humans, it was common in isolates recovered from farm animals, due to the use of virginiamicin, a related streptogamin, as a feed additive in livestock [18]. Moreover, the identification of a streptogramin A resistance gene *lsaE* in porcine MRSA suggests pigs as a possible reservoir of Q-D resistance determinants that could compromise the therapeutic use of one of the last resource drugs for human staphylococcal infections [19].

### Molecular characterization of MRSA

All 116 MRSA isolates belonged to a single sequence type, ST398, associated to two predominant *spa* types—t011 (57%) and t108 (42%)—and t1184 from a single human isolate. Nevertheless, predominant *spa* types differed between the two farms: *spa* type t011 was more frequent in Farm A (67% of the isolates) while t108 was more frequent in Farm B (75.5%) (p<0.0001). Although a wide variability of *spa* types have been associated to ST398-MRSA among pigs all over Europe, t011 and t108 are the most prevalent [15,20–22].

SCC*mec* type V was present in most of the isolates (n = 95; 82%). The remaining 21 isolates, belonging to *spa* type t108 and t1184, amplified the *mecA* gene but were negative for *ccrC*, and were therefore classified as nontypeable (NT). The large majority of NT isolates (n = 18) were recovered from Farm B. The common use of zinc oxide as a food additive in the two Portuguese farms could promote an active selection of ST398-V isolates, since *czrC* gene that promotes zinc oxide resistance was described in SCC*mec* type V of ST398 resistant isolates recovered from pigs and humans in Denmark [23].

Beta-hemolysin (81%) and gamma-hemolysin (74%) were the unique virulence determinants detected in the entire MRSA collection (Table 1), with a higher prevalence of both in Farm A (p = 0.0003 and p<0.0001, respectively). None of the isolates harboured PVL. Although the presence of virulence determinants is uncommon in ST398 lineage, a high prevalence of hemolysins was also detected in ST398 isolates from pigs in Belgium [22].

### MRSA cross-transmission

The frequent isolation of MRSA in two independent Portuguese farms might be due to animal-to-animal bacterial transmission promoted by the crowded environment in the holdings. Moreover, the spread of only two major *spa* types, t011 and t108, supports this hypothesis. The two farmers nasally colonized with MRSA (one from each farm) carried the prevalent lineages colonizing the animals in the respective farm (Fig 1), corroborating the idea that pig farming is a significant risk factor for MRSA carriage in humans [24]. Although the veterinary and the engineer are the only known links between the two farms and are both colonized with ST398-t108 strains (Fig 1), the antibiogram of the isolates colonizing both individuals were not seen among the animals (Table 1). Only a high-resolution typing method could clarify the role of the veterinary and the engineer in the transmission between farms. The household member of the veterinary was colonized with the single MRSA isolate characterized by *spa* type t1184-SCC*mec*NT. Therefore, transmission of MRSA from the swine veterinary to a household member could not be traced.

The present study showed a frequent occurrence of MRSA in two independent swine farms, highlighting the establishment of ST398-MRSA among healthy pigs, all born in Portugal. Resistance to Q-D among MRSA isolates is worrisome and should be monitored.
